# Visualisation of plastid outgrowths in potato (*Solanum tuberosum* L.) tubers by carboxyfluorescein diacetate staining

**DOI:** 10.1007/s00299-015-1748-2

**Published:** 2015-01-28

**Authors:** Wojciech Borucki, Magdalena Bederska, Marzena Sujkowska-Rybkowska

**Affiliations:** Department of Botany, Faculty of Agriculture and Biology, Warsaw University of Life Sciences-SGGW, Nowoursynowska 159, 02-776 Warsaw, Poland

**Keywords:** Plastid, Protrusion, *Solanum tuberosum*, Starch, Stromule, Potato tuber

## Abstract

*****Key message***:**

**We describe two types of plastid outgrowths visualised in potato tubers after carboxyfluorescein diacetate staining. Probable esterase activity of the outgrowths has been demonstrated for the first time ever.**

**Abstract:**

Plastid outgrowths were observed in the phelloderm and storage parenchyma cells of red potato (*S. tuberosum* L. cv. Rosalinde) tubers after administration of carboxyfluorescein diacetate stain. Endogenous esterases cleaved off acetic groups to release membrane-unpermeable green fluorescing carboxyfluorescein which accumulated differentially in particular cell compartments. The intensive green fluorescence of carboxyfluorescein exhibited highly branched stromules (stroma-filled plastid tubular projections of the plastid envelope) and allowed distinguishing them within cytoplasmic strands of the phelloderm cells. Stromules (1) were directed towards the nucleus or (2) penetrated the whole cells through the cytoplasmic bands of highly vacuolated phelloderm cells. Those directed towards the nucleus were flattened and adhered to the nuclear envelope. Stromule-like interconnections between two parts of the same plastids (isthmuses) were also observed. We also documented the formation of another type of the stroma-filled plastid outgrowths, referred to here as protrusions, which differed from previously defined stromules in both morphology and esterase activity. Unlike stromules, the protrusions were found to be associated with developmental processes leading to starch accumulation in the storage parenchyma cells. These results strongly suggest that stromules and protrusions exhibit esterase activity. This has been demonstrated for the first time. Morphological and biochemical features as well as possible functions of stromules and protrusions are discussed below.

## Introduction

Plastids are flexible organelles able to form stroma-filled tubular projections (stromules). The existence of stromules was questionable until the era of GFP technology (Kwok and Hanson 2004a; Hanson and Sattarzadeh 2008). Waters et al. (2004) found that plastid density and stromule length are negatively correlated. Holzinger et al. (2007) found that plastid projections induced by high temperatures have a considerably smaller length-to-diameter ratio than stromules and described them as protrusions. Abiotic and probably biotic stresses can induce the formation of stromules (Gray et al. 2012). However, in spite of the decades of cytological observations using different microscopic techniques, stromule functions remain an enigma. There are suggestions concerning the possible functions of stromules, such as: an increase in the plastid surface area, transportation of molecules and signalling (Kwok and Hanson 2004a, b; Pogson et al. 2008). Signal transduction realised by stromules closely associated with nuclei is under consideration (Kwok and Hanson 2004b). Improving the exchange of metabolites between plastids and cytosol realised by stromules was also postulated (Waters et al. 2004). Langeveld et al. (2000) demonstrated the involvement of plastid protrusions in the accumulation of starch. They showed the formation of the B-type starch granules in protrusions emanating from A-type granule-containing amyloplasts in developing wheat endosperm. Induction of stromule formation by sucrose and glucose also suggests the involvement of stromules in carbohydrate metabolism (Schattat and Klosgen 2011).

Stromules can be observed by means of bright field or DIC optics (Wildman et al. 1962; Gunning 2005) which proves that their presence is not the result of an intensive irradiation that is characteristic of fluorescence techniques. Substantial progress in the visualisation of stromules has been made; thanks to the use of confocal laser scanning microscopy and transgenic plants carrying plastid-localised green fluorescent protein (GFP). Photobleaching experiments using GFP-transformed plastids proved that proteins can be transported through the stromules (Köhler and Hanson 2000). However, transportation of the plastid DNA and ribosomes through stromules is rather unlikely (Newell et al. 2012). The possible exchange of macromolecules between plastids realised through stromules has recently been questioned by Schattat et al. (2012) who showed that plastids do not exchange fluorescent proteins. It has also been suggested that stromules perform all the activities of plastids except for those associated with the thylakoid membrane (Köhler et al. 1997). The lack of the thylakoid membranes in stromules was documented by Newell et al. (2012).

In this work, stromules and protrusions, two types of plastid outgrowths which differ in terms of morphology and functions, were visualised after vital carboxyfluorescein diacetate (CFDA) staining of unfixed sections. Endogenous esterases cleaved off acetic groups to release membrane unpermeable green fluorescing carboxyfluorescein. Distinct green fluorescence of the plastid outgrowths probably demonstrated their own esterase activity and enabled their precise localisation and distribution within the phelloderm or storage parenchyma cells of red potato tubers. The uptake of CFDA by intact, import-competent chloroplasts isolated from Arabidopsis plants documented by Schulz et al. (2004) as well as predicted carboxylesterase activity within these organella (Spetea and Lundin 2012) support the hypothesis that CF fluorescence expresses real esterase activity in plastid outgrowths.

## Materials and methods

Red potato (*Solanum tuberosum* L. cv. Rosalinde) plants were grown during the spring in a greenhouse at about 22–26 °C (day) or 14–16 °C (night) at the Warsaw University of Life Sciences. The maximal photosynthetic photon flux density was about 700 µm m^−2^s^−1^. Mature tubers were harvested 1 week after complete natural senescence of stems had occurred and kept in darkness at 9 °C.

### Confocal laser scanning microscopy (CLSM) investigations

Studies were conducted on fresh (unfixed) sections through the periderm of red potato tubers. Sections were examined using Leica TCS SP5II laser scanning microscope (Leica Microsystems CMS, Wetzlar, Germany) equipped with an acousto-optical beam splitter (AOBS) and an upright microscope stand (DMI 6000). A 246 × 246 µm area was imaged using a 63× objective (HCX PLAPO Lambda blue 63, 0 × 1, 40 OIL UV). The pinhole was automatically set to ‘1 Airy’.

Excitation/emission range of 561 nm/600–640 nm was chosen to visualise anthocyanin autofluorescence and 561 nm/670–695 nm to visualise chlorophyll.

### 6(5)Carboxyfluorescein diacetate (CFDA) staining

CFDA (Sigma) served as an indicator of cell viability and membrane integrity. Hand-made tangential sections of fresh potato tubers were stained with CFDA (1 mM in 0.05 M PBS, pH = 6.3) for 40 min at 24 °C. The two independent biological replicates were imaged by at least one hundred sections each. Endogenous esterase activity cleaved off acetic groups to leave membrane-impermeable CF visualised by confocal microscopy (*λ*_exc_ = 488 nm; *λ*_em_ = 515–540 nm).

### Stromule and protrusion width/length measurements, and statistical analysis

The plastid outgrowth width/length measurements were performed using confocal images. Since the outgrowths did not have sharp edges being visualised with CFDA, the full wide half maximum (FWHM) method was applied to calculate their width. The outgrowths’ widths were statistically analysed. Comparison of means was performed using Student’s *t* test, with *p* < 0.05 indicating significance. Each mean value was calculated from 30 measurements (*n* = 30).

### DAPI staining

Nuclei in the fresh material were stained with 0.5 µg/ml of 4,6′-diamidino-2-phenylindole dihydrochloride (DAPI) for 20 min and visualised by confocal microscopy (*λ*_exc_ = 405 nm; *λ*_em_ = 420–470 nm).

### Transmission electron microscopy (TEM) investigations

Hand sections of the periderm of red potato tubers were fixed according to Karnovsky (1965). The sections were then postfixed in 2 % OsO_4_ for 2 h, dehydrated in an ethanol series and acetone and embedded in glycid ether 100 epoxy resin (SERVA) equivalent to the former Epon812. Blocks were sectioned using microtomes (Jung RM 2065 and Ultracut UCT, Leica). Thin sections were collected on copper grids and stained with uranyl acetate followed by lead citrate for 1 min and examined under transmission electron microscope (Morgagni 268D). Two biological replicates were represented by ten electron micrographs each. These micrographs were analysed morphometrically to calculate the protrusions’ widths.

## Results

### Confocal laser scanning microscopy investigations

**Fig. 1:**
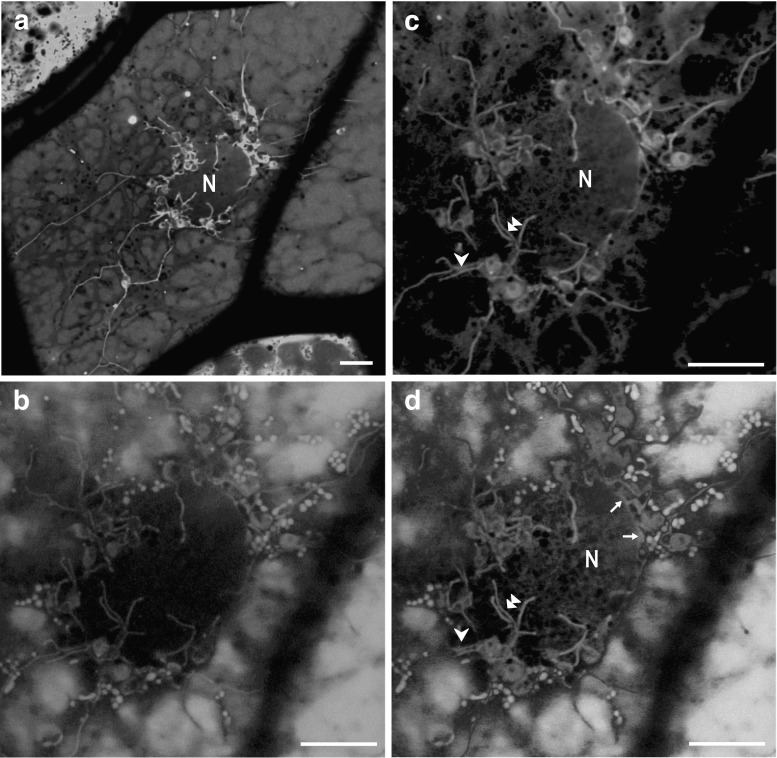
CLSM images. Stromules in the phelloderm cells of red potato (cv. Rosalinde) tubers visualised after CFDA staining. **a** General view on the distribution of stromules—merged *green* and *red* channels. Visualisation of stromules (CF fluorescence, *λ*_exc_ = 488 nm; *λ*_em_ = 515–540 nm, *false colour green*). Autofluorescence of anthocyanins located in vacuoles (*λ*_exc_ = 561 nm; *λ*_em_ = 600–640 nm, *false colour red*). **d** Stromules extend towards nucleus or cell periphery—merge of **b** and **c**. Notice that plastids form a cluster around the nucleus. Stromules extending towards the nucleus are shorter than those directed to the cell periphery. *Arrow* stromules adhere to the nuclear envelope, *arrow*-*head* bund of stromules, *double arrow*-*head* branched stromules, *N* nucleus. *Bars* 10 µm (colour figure online)

**Fig. 2:**
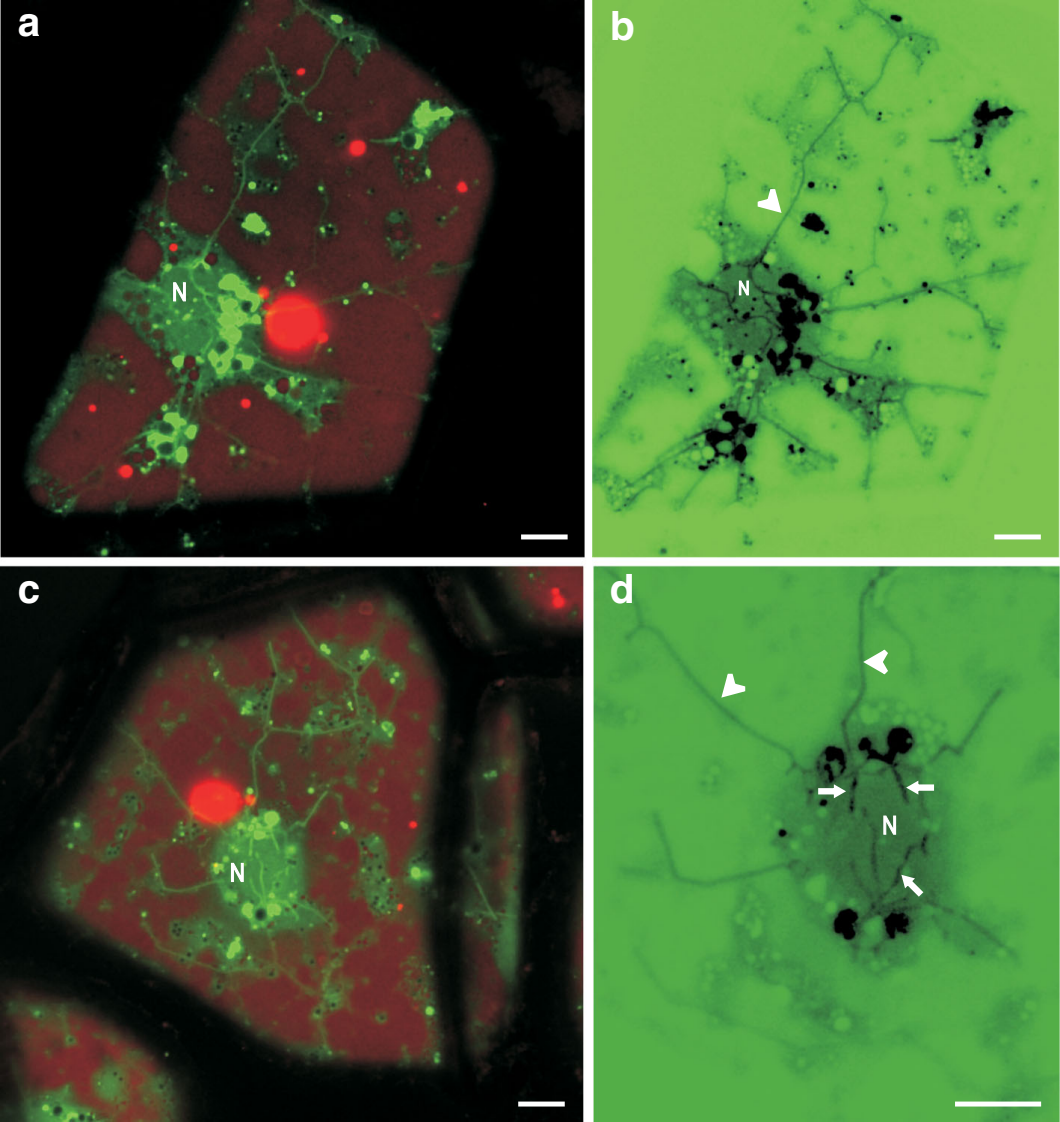
CLSM images. Comparisons of stromules’ width and fluorescence intensity in the phelloderm cells of red potato (cv. Rosalinde) tubers after CFDA staining. **a**, **c** Visualisation of stromules (CF fluorescence, *λ*_exc_ = 488 nm; *λ*_em_ = 515–540 nm, *false colour green*) and anthocyanins (*λ*_exc_ = 561 nm; *λ*_em_ = 600–640 nm, *false colour red*). Notice that anthocyanins accumulate within the vacuolar sap or inside anthocyanic vacuolar inclusions (*red* fluorescing granules). **b**, **d** Inverted *green* channels (negatives) of **a** and **b** document differences in the intensity of stromule fluorescence. Notice that stromules adhering to the nuclear envelope seem to be wider and exhibit more intensive fluorescence than stromules directed to the cell periphery. *Arrow* short stromule, *arrow*-*head* long stromule, *N* nucleus. *Bars* 10 µm (colour figure online)

The phelloderm of potato tubers was composed of 4–7 cell layers. Plastids of the phelloderm cells were clustered around nucleus (Fig. 1). In less than 5 % of the innermost phelloderm cells, distinct tubular projections (stromules) extended from the plastids. Stromules differed in longevity and direction. Short stromules (usually about 5 µm long, occasionally up to 10 µm long) emanating from the plastids were directed towards the nucleus. The stromules were in close proximity to the nucleus (Fig. 1d, see also Fig. 2d). Much longer stromules, sometimes emanating from the same plastids, grew out along cytoplasmic bands crossing the central vacuole. Some stromules extended to the cell periphery. The longest stromules were about 70 µm long. Such stromules’ width ranged from 0.2 to 0.55 µm (mean ± SD; 0.38 ± 0.12). Sometimes, several stromules forming a bunch propagating along the same cytoplasmic band could be observed (Fig. 1). Stromules that were in close proximity to the nuclear envelope were short and usually flattened (ranged from 0.6 to 0.8 µm in width; mean ± SD, 0.70 ± 0.81), and exhibited more intensive carboxyfluorescein fluorescence in comparison to the stromules extended to the cell periphery (Fig. 2). Difference between the width of both types of stromules was statistically significant.

**Fig. 3:**
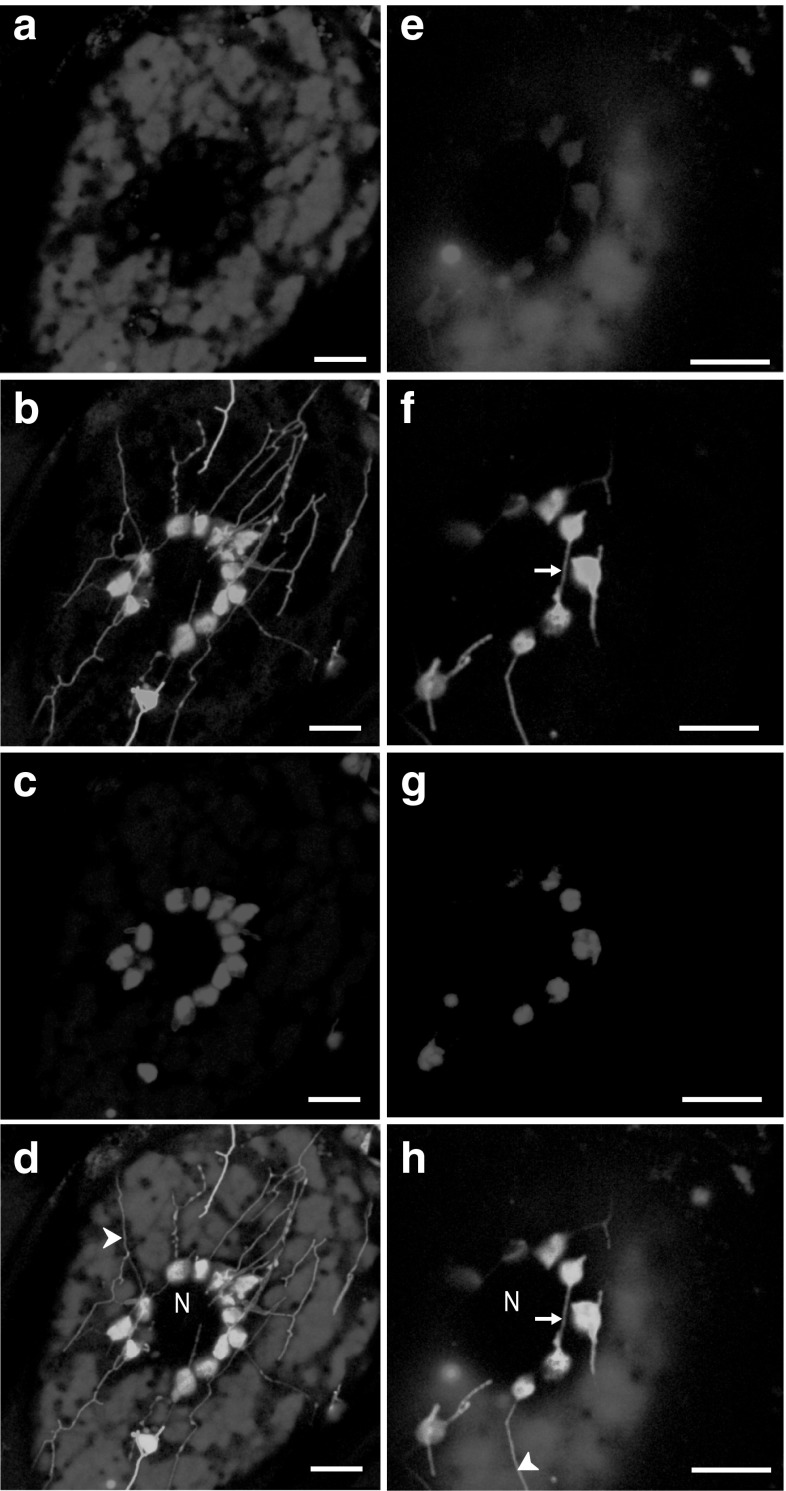
CLSM images. Visualisation of the vacuolar system (anthocyanin autofluorescence, *false colour red*), stromules (CF fluorescence, *false colour green*) and chlorophyll (autofluorescence, *λ*_exc_ = 561 nm; *λ*_em_ = 665–695 nm, *false colour blue*) in the phelloderm cells of red potato (cv. Rosalinde) tubers. **d** Merged images **a**, **b** and **c**. **h** Merged images **e**, **f** and **g**. Notice that an anastomosis between two plastid bodies do not contain chlorophyll. *Arrow*-*head* long stromule, *arrow* anastomosis, *N* nucleus. *Bars*10 µm (colour figure online)

Stromule-like anastomoses between plastid main bodies were observed. Autofluorescence of the plastid bodies characteristic of chlorophyll was revealed. Such fluorescence was absent in anastomoses (Fig. 3).

**Fig. 4:**
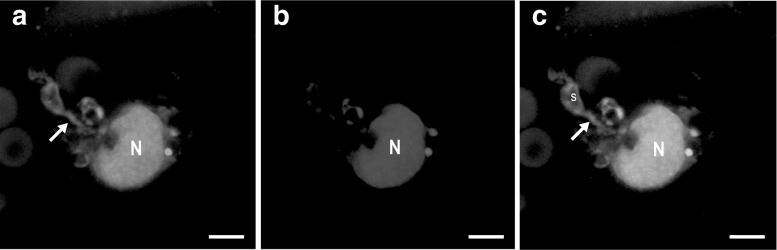
CLSM images. **a** Visualisation of protrusions emanating from an amyloplast and directed towards the nucleus in the storage parenchyma cells of red potato (cv. Rosalinde) tubers (CF fluorescence, *λ*_exc_ = 488 nm; *λ*_em_ = 515–540 nm, *false colour green*). **b** Nucleus stained with DAPI (*λ*_exc_ = 405 nm; *λ*_em_ = 420–475 nm, *false colour red*). **c** Merged **a** and **b**. Note the occurrence of ‘CF fluorescence holes’ within amyloplast body ascribed to starch granules. *Arrow* protrusion, *N* nucleus, *s* starch granule. *Bars* 5 µm (colour figure online)

**Fig. 5:**
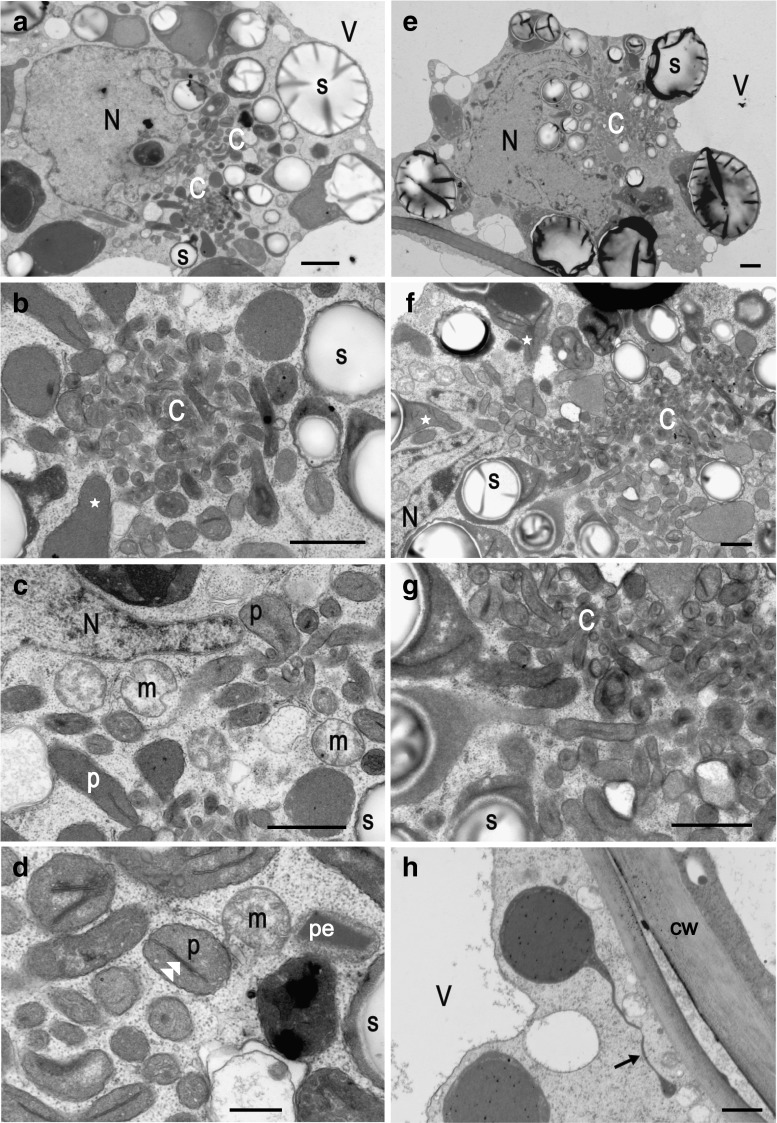
Electron microscopy micrographs. Protrusions and stromules in red potato (cv. Rosalinde) tubers. **a**–**g** Clusters of amyloplast protrusions in the storage parenchyma cells. **b**–**d** Enlarged fragments of **a**. **f**, **g** Enlarged fragments of **e**. Note that small starch granules are located closer to the cluster centre than the large ones. **h** Stromule in a phelloderm cell. *Arrow* stromule, *double arrow-head* thylakoid, *c* cluster of protrusions, *cw* cell wall, *N* lobate nucleus, *p* protrusion, *pe* peroxisome, *s* starch granule, *star* protrusion emanating from the amyloplast main body. *Bars* **a** 2 µm; **b**, **c** 1 µm; **d** 0.5 µm; **e** 2 µm; **f**–**h** 1 µm

Storage parenchyma cells possessed amyloplasts and other organelles clustered close to the nucleus. Both the amyloplasts and the nucleus had a special appearance. Amyloplast outgrowths (protrusions) concentrated in the vicinity of the lobate nucleus (Fig. 4, see also Fig. 5). The protrusions’ parts located close to the main body of the amyloplast were wider. Protrusions’ widths determined by FWHM ranged from 0.15 to 1 µm (mean ± SD, 0.44 ± 0.27).

### TEM investigations

An overview of an arrangement of the nucleus, plastids, mitochondria and peroxisomes in the storage parenchyma cells of potato tubers is presented in Fig. 5a–g. Storage parenchyma cells of potato tubers were highly vacuolated. They possessed a lobate nucleus and numerous amyloplasts containing starch granules. Amyloplasts containing the largest starch granules surrounded the nucleus. Smaller amyloplasts concentrated close to the nucleus and formed clusters (Fig. 5a, e). Larger starch granules were located at the cluster periphery, while smaller granules were located close to its centre (Fig. 5b, f). Amyloplasts’ main bodies located in the outer part of the cluster formed protrusions, which filled its centre (Fig. 5b). The protrusions directed to the cluster centre did not contain starch granules. Some protrusions got in close proximity to the nuclear lobes (Fig. 5c, f). Protrusions differed in width size (ranged from 0.2 to 1 µm; mean ± SD, 0.51 ± 0.30) with the smallest ones being located in the centre of the cluster (Fig. 5b, f, g). From one to three thylakoid-like structures were observed in the protrusions (Fig. 5d). Plastids of the phelloderm cells formed very tiny (less than 0.2 µm wide) protrusions, which might be considered to be stromules (Fig. 5h).

## Discussion

In this work, two general types of plastid outgrowths in potato tubers are described: stromules and protrusions. Stromules had been described many years ago as stroma-filled tubules, but their existence is still controversial. Transformation of plastids with GFP and the use of confocal microscopy opened up new possibilities in the visualisation of stromules. The main disadvantage of the use of GFP as a plastid marker stems from a relatively weak fluorescence intensity of the protein. Here, we described experiments on the use of CFDA staining which gave intensive fluorescence and allowed quick and extensive screening of the plant material. CF-stained stromules could easily be observed by confocal microscopy with the use of minimum laser beam power (less than 10 % of the maximal value). This may help to preserve cell structure under long-lasting observation. Moreover, the phelloderm cells are cuvette shaped, which enabled the observation of long-distance distribution of stromules, from the nucleus region up to the cell periphery, even on a single focus plane. In the case of phelloderm cells, red fluorescence of anthocyanin-containing vacuoles was an additional advantage for the spatial localisation of stromules in the system of cytoplasmic bands.

Holzinger et al. (2007) noticed that stromules were most prominent when chloroplasts were loosely arranged. Köhler and Hanson (2000) postulated that stromules are well developed when plastids are less numerous and loosely packed. Waters et al. (2004) also found that plastid density and stromule length are negatively correlated. However, in this work, most plastids of the phelloderm cells were found to be tightly clustered around the nucleus and could develop distinct stromules at the same time. Our observations indicate that three sub-types of stromules can be distinguished with regard to the phelloderm cells. Stromules of the first sub-type emanated towards the nucleus were often flattened. They were in close proximity to the nuclear envelope. This observation is consistent with a high level of contact between stromules and the nuclear envelope postulated by some authors (Kwok and Hanson 2004a, b; Holzinger et al. 2007). Therefore, it is probable that physical contact between plastids and nucleus is realised by stromules. Plastids may signal their physiological status to the nucleus, which leads to the modification of the expression of nuclear genes involved in plastid biogenesis (Kakizaki et al. 2009; Kleine et al. 2009).

Stromules of the second sub-type were directed towards distal parts of the cell, close to the cell membrane. Therefore, the stromules penetrated the whole cytoplasmic volume, which suggests that they play a role in the transduction of signals between the nucleus to the cell membrane. Such network may also allow the coordination of metabolic activity of all plastids and the remaining cytoplasm of a particular cell. Comparison of the fluorescence intensities of these two types of stromules allows us to formulate the hypothesis that stromules which were in close proximity to the nuclear envelope exhibit higher esterase activity (therefore indicating higher metabolic activity) than long stromules penetrating distant parts of the cell.

The third sub-type of stromules made anastomoses between plastids which were clustered around the nucleus and those located in distant parts of the cell. The anastomoses can also be interpreted as isthmuses between two parts of a dividing or elongating plastid. Using photoconvertible mEosFP, Schattat et al. (2012) negatively verified the concept of interplastid connectivity realised by stromules. Therefore, interconnections between plastids may, in fact, represent stromule-like structures that can differ from stromules in the origin, structure and functions. Thus, the relationship between stromules and isthmuses should be elucidated.

The normal activities of plastids take place within stromules except for those associated with the thylakoid membrane (Köhler et al. 1997). In this study, chlorophyll autofluorescence was not detected in anastomoses. But it should be underlined that the intensity of chlorophyll autofluorescence resulting from the presence of a single thylakoid in a stromule is probably too low to be detected (Köhler and Hanson 2000). Stromules substantially enlarge plastid surface area and the volume of the periplasmic space between the outer and inner plastid membranes is collectively named plastid envelope. Therefore, stromules may improve metabolite exchange between plastids and cytosol and, thus, enhance metabolic activity of the whole cell. Stromules may also prevent plastids from moving along cytoplasmic streaming.

These observations suggest that under certain physiological conditions (1) plastid metabolic activity must be uniformly distributed within the cell volume, (2) better coordination of the metabolic activity of all plastids is necessary, and (3) stromules may transduct signals from plastids to the nucleus and from the nucleus to the cell membrane. But still, physiological constraints which force plastids to form stromules need to be established.

The second main type of plastid outgrowth, referred to here as protrusions, was characteristic of amyloplasts located in the storage parenchyma cells of potato tubers. Like stromules, protrusions are stroma-filled tubular projection. But, as opposed to stromules, protrusions contain thylakoid-like structures. Whether the protrusions formed anastomoses between amyloplast bodies has not been established yet. Unlike stromules observed in the phelloderm cells, protrusions emanating from amyloplasts of the storage parenchyma do not extend towards cell periphery. Amyloplasts together with their protrusions form specific cluster(s) located adjacent to the nuclear lobes. The lobes extend towards clusters only. Such a well-defined positioning of the cluster versus nuclear lobes strongly suggests their functional relationship. It may improve plastid-to-nucleus signalling, which leads to the expression of nuclear-encoded amyloplast proteins (Rodermel 2001; Surpin et al. 2002; Strand 2004; Selga et al. 2010). What is more, Kakizaki et al. (2009) postulate that plastid protein import generates a signal that regulates expression of nuclear-encoded plastid proteins.

Protrusions exhibited low fluorescence intensity after CFDA staining, which implies that esterase activity within these structures is lower than in stromules. The presence of protrusions in the storage parenchyma cells suggests that an abundance of protrusions is linked with the relatively high concentration of sugars in sink tissues. Protrusions substantially enlarge the surface area of amyloplasts, which may improve the uptake of sugars that are later metabolised to starch. Schattat and Klosgen (2011) proved a relationship between extracellular saccharose or glucose and induction of stromules and concluded that stromules are involved in carbohydrate metabolism. It cannot be excluded that protrusions enable the formation of new starch grains in starch-filled amyloplasts. Protrusions initiate the formation of starch granules in the cereal endosperm. It was shown that outgrowths emanating from amyloplasts containing A-type of starch granules initiate the formation of new amyloplasts containing smaller, B-type starch granules (Langeveld et al. 2000). Since the phelloderm cells do not exhibit any substantial starch-forming activity, it seems unlikely that stromules formed by the cells are involved in the formation of starch granules. But, plastids outgrowths formed in the storage parenchyma cells are obviously involved in the formation of new starch granules (Fig. 5). Unlike stromules, protrusions possess thylakoid-like membranes. Protrusions were usually less than 0.5 µm long in width with the thinnest ones (~0.2 µm) concentrated in the centre of the cluster. Muñoz et al. (2008) also observed stromule-like interconnections between plastid bodies, as well as stromules in “skin cells” (phelloderm cells?) of potato tubers using GFP-targeted proteins. But the authors did not describe stromules extending towards the nucleus in the phelloderm cells nor protrusions in the storage parenchyma cells.

In this study, confocal and electron microscopy investigations showed complicated and changeable morphology of plastids in potato tubers. To our knowledge, this is the first work which documents the presence of two functionally different types of plastid outgrowths, stromules and protrusions in potato tubers. Thereafter, potato tubers may serve as a model to study plastid outgrowths. Stromules and protrusions obviously differ in functioning as only the second type is characteristic of amyloplasts and can be linked with their starch accumulating activity. Under certain, at present unknown, conditions, plastids clustered around nucleus can create a well-developed system of stromules that propagates through the whole phelloderm cells. Such system may be responsible for carrying physico-chemical signals from nucleus to the cell periphery, probably up to the cell membrane. It can be also hypothesised that stromules play an important role in balancing metabolic activities of plastids and cytosol. Unlike stromules, protrusions are permanent structures commonly observed in the storage parenchyma cells.

The results presented above strongly suggest that plastid outgrowths exhibit esterase activity. Moreover, it should be underlined that the existence of the plastid outgrowths was documented in fresh as well as a fixed material with the use of two different methods: fluorescence and electron microscopy, respectively.

### **Author contribution statement**

WB designed the experiment. WB, MB and MS-R participated in acquisition of data. WB, MB and MS-R made contribution to analysis and interpretation of data. WB prepared the manuscript and figures. WB, MB and MS-R revised the article critically. WB, MB and MS-R gave final approval to the submitted version.
